# A voice for the patients: Evaluation of the implementation of a strategic organizational committee for patient engagement in mental health

**DOI:** 10.1371/journal.pone.0205173

**Published:** 2018-10-24

**Authors:** Anna-Paulina Ewalds Mulliez, Marie-Pascale Pomey, Julie Bordeleau, Francine Desbiens, Jean-François Pelletier

**Affiliations:** 1 Department of Health Management, Evaluation and Policy, School of Public Health, University of Montreal, Montreal, Quebec, Canada; 2 Centre de recherche du Centre hospitalier de l’Université de Montréal, Montreal, Quebec, Canada; 3 International Program for Participatory Action Research on Civic Recovery, Centre de recherche de l’Institut universitaire en santé mentale de Montréal, Montreal, Quebec, Canada; 4 Institut de recherche en santé publique de l’Université de Montréal, University of Montreal, Montreal, Quebec, Canada; 5 Department of Psychiatry, University of Montreal, Centre de recherche de l’Institut universitaire en santé mentale de Montréal, Montreal, Quebec, Canada; 6 Yale Program for Recovery and Community Health, Department of Psychiatry, Yale School of Medicine, New Haven, Connecticut, United States of America; Universitetet i Stavanger, NORWAY

## Abstract

**Objective:**

There is a need for structure to achieve functional patient engagement within mental healthcare organizations, and for clarification on how to proceed on a strategic level. The aim of this paper is to shed light on the implementation of a strategic organizational structure for patient engagement in mental health by examining why and how to implement a structure, the organizational and environmental factors that facilitate or limit the process, and the perceived consequences of the implementation.

**Method:**

This paper evaluates the implementation of a strategic committee for patient engagement in a mental healthcare organization in Montreal (Quebec, Canada). The research was designed as a qualitative single case study using a deductive approach by means of a conceptual framework. Data sources consisted in ten semi-structured interviews, three focus groups, and organizational documents.

**Results:**

The strategic committee for patient engagement was implemented as a means to formalize patient partner participation, following the introduction of a vision of full citizenship. Important aspects of its implementation included its composition and role, the elaboration of a framework for patient partner participation, and finally, ongoing application and evaluation of the framework. Several facilitating factors were identified, including executive management support, leadership, and a vision behind the participation. Limiting factors mainly consisted of resistance towards patient participation and the existence of stigma. Consequences included increased and improved patient engagement, as well as reduced stigma within the organization.

**Conclusion:**

This study shows that the implementation of a strategic organizational structure for patient engagement is comprehensive. It further shows the importance of a vision and an articulate leadership involving several actors. Further research is needed regarding the impact of this type of strategic structure on a clinical level.

## Introduction

In recent years, the patient engagement approach has been studied frequently within various areas of the healthcare system. There is a consensus in the literature on the importance of engaging patients in different ways within the healthcare system to improve quality of care and positively affect other elements at both the individual and organizational levels [1, 2]. As has been frequently noted, involving patients in decision-making has to go beyond merely symbolic participation without an actual role for the patients [3, 4]. There is a need for further clarification regarding patient engagement and for a context-sensitive approach, involving several interconnected elements [5, 6].

### Patient engagement within mental health

Reviewing the literature on patient engagement, specifically within mental health, reveals additional considerations. Patient engagement is often discussed in connection with challenges linked to traditional perceptions of mental illness and the limitations that they impose on patients; for example, in terms of inequality within power-relations [7] and stigmatization [8]. When it comes to stigma, these perceptions can be expressed through negative paternalistic attitudes towards the capacity of the mentally ill to take part in decision-making [9]. Progress has been made on these fronts by incorporating patient engagement as a fundamental part of notions such as recovery and citizenship [10], two approaches aiming to promote full community inclusion and participation despite or beyond enduring negative perceptions of mental illness [11]. Recovery involves retrieving important aspects of everyday life that were lost through mental illness, even as the illness persists [12], while citizenship takes a larger perspective, encouraging patients to function as regular citizens within society, but still with due respect to the diversity they may represent collectively [13, 14]. Similarly, the “citizen psychiatry” approach, developed in France, involves active social inclusion of mental health service users [10]. In the context of Quebec, the most recent Mental Health Action Plan (MHAP) incorporates patient engagement under its goal for all to be considered citizens [15]. While the importance of patient engagement within mental health is clear, the question of how to proceed in practice needs further clarification [16–18]. There are several different models or methods for involving patients in mental health organizations, both at the individual level and at a broader level [19, 20]. This paper focuses on the latter.

### How to implement patient engagement

The literature indicates that patient engagement requires a certain support or structure in order to work well within mental health organizations, which means explicit actions need to be taken in this direction [2, 16, 21, 22]. Elements of successful patient participation that are frequently cited include actor preparation, support mechanisms, and resource allocation [20, 23]. These positive ingredients must be successfully combined, while overcoming the negative influences prevailing both within the organization and in society at large [22]. The literature provides examples of various patient engagement initiatives in mental health settings where local participatory structures have been created within organizations, such as councils and committees including patients [24, 25]. However, it is also necessary to zoom out from individual participation initiatives to adopt a larger vision of functioning patient engagement [6]. There is less evidence in the literature on establishing patient engagement structures on a strategic level to improve patient engagement across an entire organization. This knowledge gap has also been identified by Rise, Solbjør, and Steinsbekk [26], who investigated the implementation of an organization-wide strategy for patient engagement. A few studies have taken a wider organizational perspective, focusing on the establishment of a structure for the benefit of patient engagement [23, 26–29]. These studies, however, are not explicit regarding the theoretical foundations on which they rest, a key element when studying the implementation of interventions within healthcare organizations [30].

The aim of this paper is to shed light on patient engagement in mental health, more specifically, the implementation of a structure for patient engagement on a strategic organizational level. To this end, a case study was carried out. The paper will address the following research questions:

- Why and how to implement a strategic structure for patient engagement within a mental healthcare organization?
- What organizational and environmental factors facilitate or limit the implementation?
- What are the perceived consequences of the implementation?

## Conceptual framework

**Fig 1:**
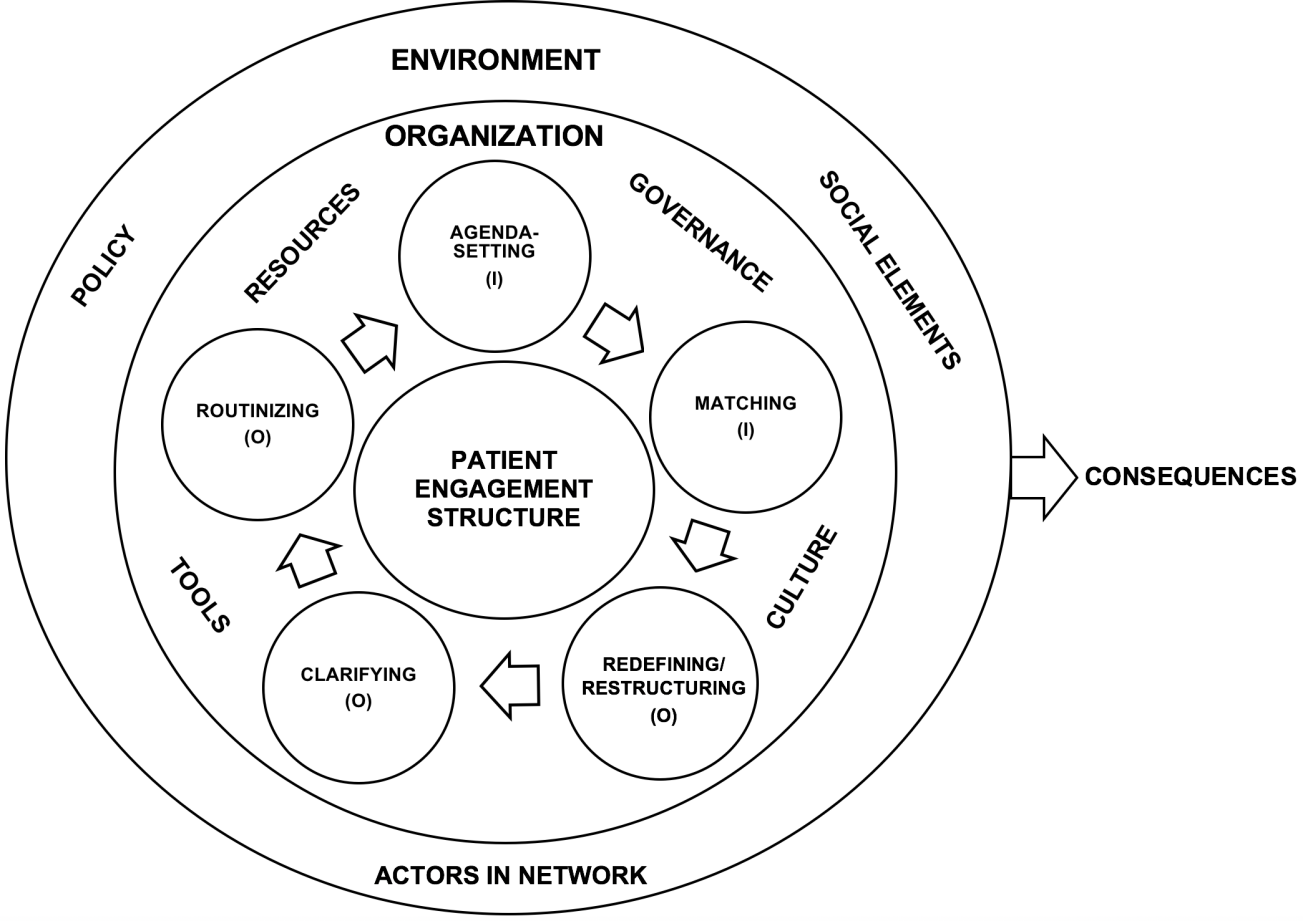
Framework to study the implementation of a strategic structure for patient engagement in mental health. The inner sphere illustrates the organization where the implementation process of the structure takes place, while the outer sphere refers to the environment of the organization. Both spheres contain factors influencing the process. The stages of the implementation process belong to initiation (I) or operationalization (O) of the structure. The consequences of the implementation are situated to the right. (Adapted from Rogers [33], Pomey et al. [35], Brooks et al. [32] and Mendel et al. [36]).

A conceptual framework was used to study the implementation process of a strategic structure for patient engagement, the influential organizational and environmental factors, as well as the consequences of implementation (Fig 1). Although there are a range of frameworks illustrating different aspects of implementation of innovation and change within healthcare [31], the body of knowledge specific to the process of implementation within mental healthcare organizations needs further development [32]. For this reason, the model by Rogers [33], which depicts the innovation process in a general way and leaves the content of each stage to be discovered, seems appropriate. Rogers describes the model as being composed of two parts: one addressing the rationale for the innovation constituting its initiation, and the other covering the actual implementation where the innovation is operationalized. In the conceptual framework of this study, the term “operationalization” is used instead of “implementation” as in the model by Rogers. This adaption has been done in order to illustrate that the implementation process consists of both initiation and operationalization. The first part of Rogers’ model includes *agenda-setting* and *matching*: identifying the need for innovation, as well as planning and designing the innovation in accordance with this need. Hence, deciding whether to continue with the subsequent stages or not occurs in the first part. The second part of the model consists of the stages of *redefining/restructuring*, *clarifying*, and *routinizin*g: mutually adjusting the innovation and organization to each other, further structuring the innovation, and finally integrating it with the organization. It should be noted that the implementation process in healthcare is non-linear [31, 34].

Factors that influence the implementation process can be either internal/organizational or external/environmental [35, 37–40]. A host of organizational factors are identified in the literature. Pomey et al. [35] categorized these factors under the four groupings of *culture*, *governance*, *tools*, and *resources*. It is also important to consider the specific context of this field and to look beyond the organization when studying the implementation of innovations within mental health [32, 36]. Environmental influences on the process of implementation include factors such as mental health policy, different social aspects of mental health (e.g., norms, attitudes, stigmatization, social exclusion) [32, 36], and various actors operating in connection with an organization [36].

## Methodology

### Study context

This paper evaluates an initiative through which a strategic committee was implemented to structure patient engagement within a large public mental healthcare organization in a metropolitan area of Montreal, in the province of Quebec, Canada. The organization provides both inpatient and outpatient services, and comprises different administrative and clinical departments. Healthcare is provided through specific clinical programs in psychiatry, as well as certain general healthcare services. The organization also conducts research and teaching activities. A strategic committee was established as a formal structure to support the participation of patient partners in different activities within the organization. The committee includes representatives from several of the administrative and clinical departments, as well as user and patient representatives. A single-case study was conducted, and various means of data collection were used to explore the implementation process of the strategic committee within the organization [41, 42]. The study was retrospective in nature, studying events that have taken place between 2008 and 2015. A qualitative approach was used to obtain multiple perspectives on the implementation process, and a deductive analysis was carried out with the conceptual framework as a base [43]. Other elements of patient engagement, such as recovery mentors [44], exist within the studied organization, but are outside the scope of this paper.

### Data collection

The data was collected through interviews, focus groups, and an analysis of organizational documents. Data collection took place between December 2015 and August 2016. A total of 27 individuals participated in the study. The participant categories were (several participants belonged to more than one category): patient partners (n = 7), a member of the users committee (n = 1), recovery mentors (n = 3), members of executive management (n = 2), researchers (n = 2), a clinician (n = 1), senior managers in departments (n = 5), middle managers in departments (n = 4), project managers (n = 2), and clinical administrative managers in clinical programs (n = 3). Ten semi-structured interviews [45], lasting approximately 60 minutes, were conducted with participants who had been involved in implementing the committee (members of executive management, managers, patient partners, researchers, one clinician, and one member of the users committee), several of whom were members of the committee. Two of the interviews involved two participants at the same time for logistical reasons. Three focus groups of 120 minutes each were also conducted as part of data collection: the first with nine actors (mainly managers, but also recovery mentors) taking part in patient engagement in different ways within the organization, the second with four patient partners, and the third with nine different actors (patient partners, managers, and recovery mentors) involved in patient engagement and, in most cases, in implementing the committee. An objective of the first focus group was to narrow down the scope of the research project, while the last group also served to validate the research team’s understanding of the previously collected data. Guides for the interviews and focus groups were developed, then validated during meetings of the research team. The interview guide was modified as deemed appropriate after the first interviews carried out in December 2015 [46]. All guides were based on the same themes from the conceptual framework (stages of implementation, factors, and consequences), allowing for the process to be examined through various perspectives. Finally, an analysis was conducted of relevant organizational documents (annual reports of the organization, organizational charts, newsletters, various publications, annual reports of committees, an evaluation report concerning patient partner participation, meeting minutes, presentations, and working documents). The collection of diversified data allowed for triangulation [41, 43].

#### Participant recruitment

Participants were recruited with the help of the research team’s main contacts at the organization. An initial list was assembled, containing participants who could provide information on the studied events. As data collection proceeded, further participants were recruited through the “snowball” method as long as they were vetted as being able to provide significant contributions [43]. Participants were contacted via telephone or e-mail by a member of the research team, except for the participants of two of the focus groups, who were invited by contacts at the organization.

### Data analysis

The interviews and focus groups were recorded and transcribed, and all data were analyzed following the iterative process of Miles and Huberman for qualitative studies [46], using the qualitative data analysis software package QDA Miner 4.0 [47] (see Table 1). The first author coded and compiled the data, while the second and fourth authors also coded two interviews and one focus group to validate the attribution of codes. All authors verified the results emerging from the analysis.

**Table 1:** Presentation of the data analysis process.

Elements of the data analysis process	Description
**Development of codes (software)**	The conceptual framework and research questions were used to create the initial codes of the coding tree: Implementation process (different stages) Facilitating and limiting factors Organizational (culture, governance, tools, resources) Environmental (policy, social elements, actors in network) Consequences
**Attribution of codes (software)**	Coding of organizational documents, and transcribed interviews and focus groups
**Modification of codes (software)**	Changes made throughout the analysis and discussed during the meetings of the research team (e.g., creation of sub categories to factors and consequences)
**Combination of codes (software)**	Combinations of codes for facilitating and limiting factors and implementation stages allowing for the moment of influence to be identified (see S1 Appendix)
**Overview of data (software)**	The coded data segments were highlighted and grouped together (e.g., all events of each implementation stage, all factors of each category)
**Summaries and chronology**	Creation of: Report summarizing the implementation process Table containing key events and factors (S1 Appendix) Chronology of key events during 2008–2015

### Ethical considerations

Ethical approval was obtained from the University of Montreal Health Sciences Research Ethics Committee (14-127-CÉRES-D), and from the ethics committee of the Centre hospitalier de l'Université de Montréal (MP-02-2015-5710—CE.14.232), which handled the multi-site certification of the research project. Written informed consent was obtained from all participants of the study.

## Results

The conceptual framework is used as a foundation to present the results, starting with the five-stage implementation process of the strategic committee, followed by organizational and environmental factors of influence, and finally, the consequences of implementation.

### Implementation process of the committee

The first two stages of the implementation process (agenda-setting and matching) contain two overlapping chains of events leading to the creation of the strategic committee, while the following three (redefining/restructuring, clarifying and routinizing) cover the establishment and activity of the committee.

#### Agenda-setting (2008–2011)

The first stage of implementation involved launching patient partner participation within the organization (the term patient partner is used for patients participating in various activities). This included identifying the need for a formal structure and opportunities for realizing it.

This stage saw the beginning of two overlapping chains of events. First, following the inclusion of the notion of *recovery* in the Quebec MHAP for 2005–2010 [48], which entails patient participation at an organizational level, the management of one clinical department took the initiative of involving patients in the design and the organization of services. A reflection regarding recovery and how to go about involving patients was undertaken within this department, together with actors from the research department. The process was described as “action-research”, as the ongoing reflection was complemented by initial experiences of patient involvement. Many questions were raised:

*“[…] ‘who do I invite*? *Why do I invite them*? *Should they be paid*? *How will the others react*?*’ So*, *we were stuck with all these questions*, *but we really did it sort of as ‘action-research’*. *We told ourselves*: *‘Well*, *we’ll figure it out*, *we’ll start integrating them*.*’”* (Interview 75, non-patient)

Actors in the above-described reflection wanted the participation of patient partners to work well, in addition to capturing and leveraging their experience as patients when involving them. The initiating department organized training for personnel and patient partners, in collaboration with a provincial mental health association with expertise in involving patients. Furthermore, a partly independent self-help group run by patients was formed to discuss recovery and help develop the participation of patient partners. This self-help group also constituted the pool of patient partners at the time, which implied them participating in different activities within the organization.

Thus, a structure for participation began to take shape, which included developing a policy and arranging for patient partners to be compensated for their time. However, it was recognized that the process would have to be further formalized, including evaluation, to function well, as ethical concerns were raised. Indeed, with regards to the psychological safety, getting engaged as a mental health patient in discussions on patients’ experience could sometimes rekindle painful memories. While assuming leadership and responsibility, the involved actors were aware that the engagement procedure should not and could not be improvised given the identified psychological risks.

*“There were already patients that were integrated within patient-partnership activities*, *within committees*. *They felt a need to formalize all of that…”* (Interview 73, patient partner)

The second chain of events concerned actions at a strategic level that took place starting in 2010. Key actors in the organization created an opportunity to set up a formal structure for participation, making it an institutional priority. A member of the research department–already involved in discussing patient participation–introduced a new angle to recovery by combining it with another approach: *citizen psychiatry*. This inspired a member of executive management (the deputy CEO, who became CEO later in the process), who had already identified a need for an overall organizational change, to push for a transition away from traditional functioning towards becoming a university institute. Observing that the recovery approach alone did not work in the context of Quebec, an effort was led to develop a concept combining elements of both citizen psychiatry and recovery.

*“Recovery*, *which is an American movement to which the francophone milieu did not at all adhere*, *zero*…*”* (Interview 72, non-patient)

Indeed, although there was an openness to the underlying values and principles of the English-American concept of recovery, one of the core priorities of the MHAP, there was a need to adapt it to eventually apply it in a French-speaking area in Montreal.

#### Matching (2010–2011)

This stage involved exploration concerning recovery and citizen psychiatry, leading to the creation of the strategic committee for patient engagement. Several key actors from previous discussions (from the initiating department, the research department, and executive management) worked together on how to develop the notions of recovery and citizen psychiatry within the organization. This investigative work involved deliberation, observation, and conceptualization presented subsequently in the three following paragraphs.

These key actors organized a civic forum to discuss recovery and citizen psychiatry. The forum consisted of presentations and discussions involving different participants: patients (some of whom were also presenting), executive management and other employees, community organizations, patient organizations, and international actors. It led to a deeper exploration of the subject and increased collaboration both inside and outside the organization.

After the forum, field research visits were organized to observe examples of recovery and citizen psychiatry. To see how services could be dispersed throughout the city instead of being confined to specialized settings, a trip to France was organized, with a wide range of participants. This trip figured prominently in the collected data. Patients (n = 3), researchers (n = 2), managers, including executive (n = 4), clinicians (n = 2), project managers (n = 3), and family members (n = 2) spent one week studying the French implementation of citizen psychiatry. While the first stop of the trip involved observing the approach within an organizational setting, the second stop provided an inspirational look at the integration of patients within the community. However, much of the significance of this trip derived from the experience of travelling together. The equality between all participants was described as eye-opening, considering how far from equal the relationship between patients, health professionals, and other personnel was in the everyday functioning of their own organization.

*“*. *it was completely transformative…*, *we lived for a week together*, *all of us*. *[…] For a week*, *we ate all our meals together*, *we slept at the same place*, *we spent our days together*, *we waited for the train together*. *It really changes the relationship with*… *even with the professionals*, *the physicians*, *all of that*.*”* (Interview 71, non-patient)

Following this experience, the previously mentioned key actors conceived an approach adapted to their organization. A new clinical vision centered on full citizenship [49] was formally included in the organization’s strategic plan to promote full community inclusion and participation, in decision-making and planning processes of the institution as well as in collaboration with the surrounding community and other partners through civic forums. The vision was full citizenship for all, founded on the participation of patients at various levels.

*“…the thing about recovery centred on the individual*, *and the more public citizen approach*, *is that we can support individuals in having an influence on social determinants and public action*. *That is how we combine them*.*”* (Interview 70, non-patient)

With the introduction of this vision, initiatives to involve patient partners in different activities increased across the organization. The same member of the research department was given a mandate from executive management, along with funding, to realize the participation of patients according to the new vision of full citizenship.

At this point, the two chains of events converged, i.e., the identified need for a formal and safer structure for the participation of patient partners within the organization, and the opportunity to create it (see the agenda-setting stage). The mandate for realization of patient engagement took concrete form through the creation of an organizational committee to frame participation in line with the new vision.

*“We involved patients*, *but at the same time… we had a bunch of questions for which we did not have answers*. *So*, *at one point*, *there was a need for a space to reflect on this properly*, *and since it had worked well in the beginning*, *we told ourselves*: *‘If we want it to last*, *we need to create an official committee’*. *To have a budget*, *for it to not go off in all kinds of directions*, *for people to feel good about participating*, *and also… so that we weren’t doing patient partners just because it’s good for institutional appearances*.*”* (Interview 75, non-patient)

#### Redefining/restructuring (2011–2015)

This stage involved establishing the strategic committee for patient engagement and determining its role. It should be noted that the events and actions of the two last stages of implementation (clarifying and routinizing) also took place during the same time period as the current stage, 2011–2015.

The committee was described as developed “in action”. The collaboration between two key actors continued (from the initiating department and the research department), as they took on leadership roles as co-chairs of the committee in the beginning. The composition of the committee gradually evolved as other representatives were invited, to finally comprise eight members. The committee’s representativeness was discussed; there was a desire to have a wide range of perspectives from within the organization to better promote the participation of patient partners. To this end, the committee included members from several different departments (research, support for social integration, teaching, clinical services) and from the organization’s users committee, while a patient partner was assigned to the role of coordinator. This patient partner was also co-director of a user-led private non-profit organization created in parallel with the committee, where patient partners promote and transact their experiential knowledge through, among others, participatory research. Eventually, another patient partner was recruited, who was also a member of the previously created semi-independent self-help group (this person subsequently also joined the board of directors of the non-profit organization).

*“We had decided that ideally we should have two patient partners on the committee*, *so that people would not feel intimidated*, *would feel that there is a peer that can understand them*.*”* (Interview 73, patient partner)

Among the members from different departments were two clinical psychologists by training as representatives of clinicians, however, at the time, they were assigned to clinical-administrative work rather than clinical work per se. A few members changed over time, and a fresh perspective was ensured by regularly rotating the committee chair.

*“This committee changed chairperson every two years to ensure that the concept spread out in all the departments*.*”* (Interview 81, non-patient)

The committee was linked to and reported to executive management.

*“We deemed*, *within the committee*, *that it was necessary to have a direct link to executive management*. *For the committee to be transversal within the institute*, *not dependent on one particular program*.*”* (Interview 73, patient partner)

The first meeting of the committee took place in the Fall of 2011. Meetings were held six times per year on average. In the beginning, the committee focused on formulating its mandate. Its aims were to promote, document, and evaluate the participation of patient partners, and to establish a policy for participation. This included serving in an advisory capacity to executive management. The committee had a broad role, described as both supervisory and consultative, functioning both as a “watch dog” and a “guide”, aiming to support patient partner participation within the organization. The role was amended as the work of the committee progressed.

Each year, upon request from the committee, executive management allocated a budget for the compensation of patients participating within the organization (ranging from CAD$5,000 in 2011/2012 to $15,000 in 2014/2015). The hospital’s foundation provided additional funding, covering costs beyond the actual participation of patient partners such as transport to activities taking place outside the organization.

#### Clarifying (2011–2015)

Using the previous, more informal structure for the participation of patient partners as a starting point, the committee proceeded to articulate how it should be framed in the light of full citizenship. This stage involved two main tasks: the creation of functioning conditions for the participation of patient partners, and communication and promotion.

The committee developed a centralized system as a framework for participation activities by establishing: 1) A formal policy, including practical functional procedures for participation, which were illustrated in a chart (a document detailing the different steps and the roles of the actors involved). The policy had already been elaborated before the formalization of the committee, but was revised and adjusted at this point. 2) Standard forms for requesting the participation of a patient partner and for compensation. These were made available through the intranet, to be used by different actors within the organization wishing to involve a patient partner in an activity. 3) A tracking document for closer monitoring of participation activities and related compensation, which was created as part of budget management.

The committee’s work was characterized by ongoing reflection and discussion. Clarifications were made along the way, for example, regarding aspects of the procedures, the terminology used (patient instead of user, indemnity instead of payment), and the budget (compensation amounts, attribution guidelines, and fiscal considerations). The committee also sought to understand the functioning of patient partner participation, through internal research and collaboration with research projects within the organization. There was a desire for participation to function well, and for patients to not just go through the motions and receive a cash indemnity.

*“Of course*, *for us to approve a patient-partnership activity*, *it had to promote the full citizenship of the users involved […] and take advantage of their experiential knowledge*.*”* (Interview 73, patient partner)

*“Although included in the accreditation*, *putting a patient on a committee doesn’t mean anything*. *[…] There has always been a preoccupation*, *we’ve always done a follow-up*, *with the patients*, *and with the manager or clinician initiating the committee…”* (Focus group, non-patient)

Different challenges emerged along the way, leading to the committee reflecting on ethics. This concerned, for example, the timing of the indemnity (direct or delayed) so that the motivation to participate was not linked to satisfying primary needs, and how to act when a patient partner seemed to be in difficulty when participating.

*“And what we saw is that at times when people*, *users*, *spoke in public*, *they were stressed*, *since some people are nervous*. *Sometimes to the point that they seemed lost*, *they no longer made sense*. *At that point*, *we*, *in the committee*, *asked ourselves*: *‘What do we do in these types of situations*? *Do we take away the microphone*, *something we would not do with a psychiatrist […]*?*’ So*, *the committee would think about these challenges*.*”* (Interview 70, non-patient)

More functional aspects were also dealt with, for example patient representativeness, an issue that arose when the same patients were assigned repeatedly. This was a concern raised by several interviewees.

*“We need to switch patient partners*, *because otherwise we will always come back to the same viewpoint […]*. *That’s what worried me a little more*, *since it’s like with professionals*, *we have our professional views*. *If it’s always this same user on all the committees*, *well*, *it’s their experience*, *but it’s not representative of others…”* (Interview 74, non-patient)

In line with the vision of full citizenship, the committee wanted to make the role of the patients more equal to those of other actors within the organization. It brought attention to physical places and activities where patients were not allowed. One example, pointed out by a patient partner on the committee, was lack of access to the documentation centre. This struck the committee as paradoxical, since the patients that participated in various activities also needed documentation resources. The experience also highlighted the important role of the patients.

*“Because we don’t see it anymore*. *It takes patients to denounce it or to bring attention to things like that*.*”* (Interview 70, non-patient)

The committee members informed the rest of the organization about their role, sharing policies and procedures. Annual reports were produced to summarize their activities and accomplishments, as well as the various participation activities taking place within the organization. They also promoted both their own work and the participation of patient partners, for example through presentations at conferences, orientation days for new employees, and promotional banners. These types of activities also aimed to “destigmatize” mentally ill persons. Promotion took place both inside the organization and, increasingly, on the outside, extending even to exchanges with international actors. Patient partners, both members of the strategic committee and others, were involved in all of these activities.

#### Routinizing (2011–2015)

During this stage, the procedures for patient partner participation were applied on an ongoing basis, an evaluation mechanism was created, and participation continued to increase.

The participation activities within the organization passed through the committee and followed the procedures they had established. At this point, the procedures for participation, which had been developed and gradually improved by the committee, were described as well- functioning.

*“It was integrated into the whole system*. *Whether it was a public conference*, *a management committee on the organization of services*, *the welcoming of employees*, *in many areas*. *It was logical*, *it was natural to say that a patient partner was integrated […] ‘did you pass by the patient partner kiosk*?*’ It was a reflex*.*”* (Focus group, non-patient)

The framework for participation activities relied on the ongoing administrative work of a few members of the committee, including the coordinator, and consisted in handling the procedures for participation: 1) When a health professional or manager within the organization wished to involve a patient in an activity, a request form was filled out on the intranet and automatically transferred to the committee. 2) The request was processed (approved or refused), and 3) for approved cases, a patient partner, either chosen by the committee or identified by the person making the request, was assigned. Many patient partners came from the self-help group and the user-led private non-profit organization, but health professionals and managers also referred patients. 4) Preparation for participation (e.g., reading material) was handled by the person requesting the participation, with the committee available for support. 5) After the participation, the committee received satisfaction surveys filled out by both the patient partner and health professional or manager, for follow-up. These surveys provided insight to the committee on the participation and how it could be improved so that patients could truly take part in the activities. Patient partner compensation and the administration of satisfaction surveys were handled by executive management in the beginning, but were eventually taken over by the committee coordinator, who also was a certified recovery mentor paying particular attention to the comfort of patient partners in their engagement.

Based on these procedures, including the satisfaction surveys, an evaluation mechanism was developed. The committee formed a sub-committee (which included both patient partner members) responsible for evaluating the satisfaction of health professionals, managers, and patient partners involved in participation activities. In addition to the satisfaction surveys, focus groups were carried out. This provided feedback regarding different aspects of the procedures and the participation itself. For example, a need was identified for better preparation prior to participation activities.

Although policy and procedures were developed, certain aspects remained informal, for example, the criteria for recruitment of patient partners.

The participation activities, which took place both inside and outside the organization, touched many different areas: organization of services, research, teaching, evaluation, and others (see Table 2).

**Table 2:** Examples of participation activities.

Area of participation	Examples of activities
**Organization of services**	Testimonies on specific topics Clinical programing committees Other committees (suicide prevention, legal psychiatry, etc.) Writing of guides (practical, welcome) Workshops
**Research, teaching, evaluation**	Research projects Validation of research instruments and surveys on service satisfaction Course for medical students
**Others**	Conferences and presentations Civic forums Study trips Video clips aiming to reduce stigmatization “Living libraries” where patient partners tell their stories Theatre pieces

The level of patient participation within the organization facilitated through these procedures doubled from 2011 to 2014. Both the number of activities and the number of patient partners increased (27 activities and 30 patient partners in 2011/2012 versus 60 activities and 67 patient partners in 2013/2014). The activities often involved several patient partners at the same time, and could be punctual or take place over several occasions. More and more actors within the organization requested patients for participation, and resistance towards the practice lessened, as reported by several interviewees. However, patient partners also expressed a wish for more participation.

This positive trend ended with the arrival of a reform of the Quebec healthcare system in 2015 [50], leading to the organization merging with others. Before the reform came into force, a decrease in the number of participation activities was observed, and a discussion began on how to ensure continued activity of the committee and how to spread this strategic patient engagement practice over a larger scale within the new organizational structure.

### Facilitating and limiting factors

Several organizational and environmental facilitating and limiting factors were identified, as described below (see Table 3).

**Table 3:** Facilitating and limiting factors.

Factors	Facilitating	Limiting
**Organizational**	Executive management support Management support in initiating department Leadership by key actors from executive management/research department Shared leadership in management-research Vision of full citizenship “Standard-bearers” of the vision Financial resources	Resistance within organization Difficulty in sharing the vision
**Environmental**	Mental health policy (MHAP 2005–2010) Collaboration with self-help group International influence Supportive network for participation Obligation to evolve in research and innovative practices	Stigmatization Reform in healthcare system (2015)

#### Organizational factors

A key *facilitating organizational factor* was continual support from executive management throughout the initial development of patient partner participation, the elaboration of the full citizenship vision, and the establishment of the committee. This support was realized effectively by keeping the committee connected to executive management and allocating a budget for their activity. Management support within the initiating department was also determinant for the start of patient partner participation.

*“… we were supported by executive management*, *by our managers […] also by his own boss the CEO at the time*. *You cannot sustain something that complex*, *overcoming resistance*, *if it is not endorsed by executive management*.*”* (Focus group, non-patient)

*“The whole board of directors of the hospital was inspired by these new ways of doing things*.*”* (Interview 71, non-patient)

Strong leadership and dedication from the key actors in executive management and the research department were essential in elaborating the vision of full citizenship. There was also a shared leadership and understanding (“common philosophy”) among the initial members (the co-chairs) of the committee, which facilitated its creation through a collaboration of management and research.

*“*…*So*, *we joined forces to be able to create this unity to sustain the process*.*”* (Focus group, non-patient)

Furthermore, the vision of full citizenship enabled a cultural change within the organization, which helped the establishment of the committee and its work.

*“… what is important*, *is what made it work*. *We were supported by executive management*, *it was implemented in the organizational culture*, *we talked about full citizenship*, *there was a clinical vision accompanying these activities…”* (Focus group, non-patient)

The committee’s work was also facilitated by individuals who became “standard-bearers” of the vision of full citizenship within the organization (many of them having participated in the eye-opening trip to France), for example psychiatrists who referred patients to be patient partners, as well as key actors within the committee. Finally, financial resources made it possible to start the committee and for it to continue functioning over the years.

*Limiting organizational factors* included resistance from certain actors within the organization, mainly managers and health professionals, particularly at the beginning of the participation of patient partners.

*“There was also a little bit of insecurity in integrating the patients*, *because some people–not all*, *but some–thought that we were there to slow down their work*.*”* (Focus group, patient partner)

A final factor was the difficulty in sharing with the rest of the organization the vision of full citizenship that had been conceptualized by a few key actors.

*“…the less you communicate*, *the more people in the field will say*: *‘It’s management style*.*’ The more frequent the communication*, *the clearer it is*, *that will help*. *And to explain what full citizenship meant*, *because we did not understand it in the beginning*. *‘What does it mean*?*’”* (Interview 74, non-patient)

In order for employees to align with the vision, the importance of ensuring that they understand its implications was pointed out.

#### Environmental factors

In terms of *facilitating environmental factors*, mental health policy (MHAP 2005–2010) instigated the process of involving patient partners within the organization. There was also influence from international actors in recovery and citizen psychiatry, both regarding the full citizenship vision and means for involving patient partners.

*“We came back home inspired by all these values and*, *for a long time*, *we would invite patients to be part of our committees […] where the experiential knowledge is important*, *because the patients were sometimes bored when it did not concern them*, *I think*.*”* (Interview 71, non-patient)

The self-help group was an important collaborator and provided support throughout the implementation of the committee, from the first steps in patient partner participation. Similarly, the supportive network for patient participation both inside and outside the organization (e.g., user-led private non-profit organization, self-help group, community organizations where patient partners were active), contributed to the committee’s work. Additionally, the aim of becoming a university institute, which entailed evolving towards a structure of research and innovative practices, certainly influenced the process.

*Limiting environmental factors* mainly consisted in the existence of stigma, which was expressed within the organization through resistance towards the participation of patient partners and the existence of discriminatory organizational structures. The enactment of the 2015 healthcare system reform in Quebec also affected the committee’s activity.

### The consequences of the implementation of the strategic committee

According to interviewees, implementation of the committee for patient engagement made ongoing patient partner participation possible, by creating a formal structure for it within the organization, including a budget for participation and the production of annual reports. The evaluation mechanism that was instituted enabled the committee to obtain feedback and improve the procedures for participation, for example by strengthening support for actors involved in participation activities.

The increased participation of patient partners was due not only to the vision of full citizenship, but also to the existence of the committee, which promoted and supported participation in a systematic way. This generated a change in culture within the organization, which reduced resistance to patient participation. By including members from various departments and actively advocating broader participation, the committee also contributed to increasing the scope of participation within the organization.

*“The involvement of [name]*, *then the research*, *as well as the teaching department to spread the practice*, *helped make it [patient engagement] sustainable*.*”* (Focus group, non-patient)

Several discriminatory structures within the organization were removed through the observations and actions taken by the committee. With the committee’s work and increased participation activities, patients were given a formal and more significant presence within the organization, which contributed to destigmatize mentally ill persons.

*“… when it comes to stigmatization*, *what works is direct contact*. *Of course*, *we try to provide guidance through several other initiatives such as conferences*, *information pamphlets […] But*, *for now*, *researchers have concluded that what works is direct contact*.*”* (Focus group, non-patient)

## Discussion

### Limitations of the study

As data collection was carried out retrospectively, no observational data on the implementation process was included in this paper, which is an important part of case studies [41]. Many years had passed since several of the studied events, and due to the 2015 reform of the Quebec healthcare system, which involved structural organizational changes, including the reorganization and elimination of various managers positions [50], not all concerned informants could be included in the study. Additionally, at times, the embeddedness and parallelism of different events in the committee implementation process led to difficulties with regards to knowing when an aspect was dependent on the committee itself or on the vision of full citizenship. Finally, the perspective of health professionals could have been included in this study in greater depth.

### Main findings

The results show how a structure for patient engagement, in the form of an organizational committee on a strategic level, can be put in place. The committee was implemented to formalize the already-existing participation of patient partners, as a new vision of full citizenship was introduced, which implied changes in the organization. Implementation of the committee progressed through stages, from its initial formation and structure, to elaborating a framework for patient partner participation within the organization, and finally to the ongoing operation and evaluation of the framework. Several facilitating factors were identified, including executive management support, strong leadership, international influence, and guiding vision for participation. Limiting factors consisted mainly of resistance towards participation and the existence of stigma. The implementation of the committee led to increased and improved patient partner participation. It also helped reduce stigma within the organization, by contributing to a more formal and widespread presence of patient partners. The results of this study confirm that implementation of a strategic committee for patient engagement is a multidimensional, complex process and raises several considerations. A few of these are discussed below.

### The committee as a model for improving patient engagement

Despite the lack of literature on the subject, both in mental health specifically and in other healthcare domains, the need for strategies at the organizational level to spread the practice of patient engagement has been emphasized [23, 26, 51]. In the present study, successful realization of such a strategy was made effective by ensuring representation from various departments on the strategic committee, and by advocating more extensive participation of patient partners. This suggests that the committee could serve as a model for others interested in implementing a similar structure. Also observed was the kind of active and deliberate leadership to improve patient engagement that has been identified as a requirement in the literature. This leadership includes promotion of the approach and support for its realization, both in practical terms and from an ideological standpoint, relating to the values of the parties involved [21, 52]. This underlines the need for the explicit creation of a structure for patient engagement that is both practical and democratic [25]. The present findings agree with previous studies examining efforts to take patients’ experiential knowledge into account [21, 25, 53] and to reduce visible discriminatory elements [7]. However, a change in culture is required [7], as we discuss further down. The visible and influential role of coordinator of the strategic committee held by a patient partner could be considered as a concrete manifestation of democratization and inclusion of experiential knowledge. It can also be seen as an expression of recovery and greater participation within the healthcare system and society–as has been promoted in the Quebec context for many years through mental health policy [15, 48]. As such, it exemplifies the many facets of individuals, ranging from patients to citizens [54].

Based on the literature, two limitations of the model for patient engagement presented in this study can be identified. The first is the non-inclusion on the strategic committee of healthcare professionals doing clinical work. A literature review on patient engagement in change management pointed to a lack of focus on healthcare professionals as leaders of the approach [55]. The second limitation concerns the lesser importance granted to preparation for participation. The importance of training to work together and take part in new initiatives, on a scale greater than that of an individual participation activity, has been expressed [56].

### A vision behind patient engagement, and articulate leadership

The vision of full citizenship in this study gives conceptual meaning to the participation of patient partners [57], responding to calls for conceptual clarification regarding patient engagement [58]. It has been argued that, for successful transmission, it is necessary to have not only a conceptual vision to act as a vehicle for change, but also a manifestation of this mental content by actors within the organization [59]. Thus, having a vision supporting the participation is not enough on its own. In this case, there was an initial lack of comprehension lower down in the organization, before organizational culture change occurred. Leadership is an important element of organizational culture change [60], which requires leaders who can grasp the essence of an innovative vision and translate it into action, especially within organizations in fields such as mental health [61]. Building on this, the present study agrees with previous research recommending that patient engagement should be given a formal place within the overall organizational culture of mental health organizations [55].

### Characteristics of the implementation process

The results also confirm the non-linearity of the implementation process in healthcare [31] as well as of patient engagement [6], as illustrated by the changing composition of the committee for example, or the successive modifications to the procedures for participation. Non-linearity was further expressed in each of the last three stages of implementation of the committee, which ran in parallel and overlapped. Findings like these confirm that implementation within healthcare is an open-ended process, continuing throughout the innovation’s existence [62]. Furthermore, the committee’s implementation process was characterized by collective action involving a variety of different actors, a suitable approach for tackling various complex subjects arising along the way [63]. Patients participated in each implementation stage of the committee, which is a key element when it comes to changes in mental healthcare organizations [55, 64] and challenges the perception that mental health patients may not have the capacity to participate in decision-making within organizations [16]. Finally, this study supports and illustrates the importance of constant management support, as brought up in the literature on both implementation [39] and patient engagement [23].

Using a conceptual framework helped highlight several aspects of the implementation process of a structure for patient engagement in a mental health context. Drawing on Rogers [33], the framework of this study distinguishes between initiation and operationalization, allowing for exploration of the preparatory actions leading to the implementation of the committee. The large role played in this case by the vision of full citizenship, which was developed in the initial stages, confirms the importance of these first steps as mentioned in the literature [65]. To further develop the conceptual framework based on the results of the present study, the consequences of implementation could be categorized in terms of quality, range, and volume of participation, as well as organizational culture.

### Continuing implementation process

As the results indicate, the routinizing stage of the implementation process was affected by the reform of the Quebec healthcare system, spurring reflection on how to pursue the committee’s activity within the new larger organization. Executive management support for patient engagement within the larger organizational entity, as well as the continuing participation of key individuals from the implementation of the original structure, have been raised as key elements in another case described by Pomey et al. [66] concerning the successful propagation of a similar structure for patient engagement following the Quebec healthcare system reform.

## Conclusion

This paper examines the implementation of a committee for patient engagement across an entire organization. It shows that such a structure’s success in increasing and improving patient engagement depends on many factors and that multiple dimensions must be considered. For patient engagement to operate well, broadly, and in democratic conditions within mental health organizations, the implemented structure must be guided by a strong vision combined with articulate leadership provided by diverse actors. Given the above, some recommendations for decision-makers interested in implementing this type of structure are to include multiple actors in the process, to have a clear vision behind the change, to appoint leaders, and finally to assign adequate resources so that all aspects of patient engagement within the organization can be monitored.

The usefulness of a conceptual framework is brought forward in this paper by confirming that existing theory can be used to explain our case study. Theoretical insights are also provided through the development of categories regarding the consequences of implementation. Finally, a better understanding of the implementation process is provided through concrete presentation of the events at each stage and the factors with influence.

The reform in the healthcare system of Quebec, which took effect in 2015, has raised several questions. What do these types of changes entail for organizational structures for patient engagement such as the committee in this study? What are the conditions for successfully propagating a structure for patient engagement in a larger organization? These are worthwhile topics for additional research. Moreover, this kind of strategic organizational structure for patient engagement requires further research to explore its impact on quality of care and services on a more clinical level.

## Supporting information

S1 Appendix The implementation process of the committee and influencing factors. (PDF)
