# Supplementary material for: A voice for the patients: Evaluation of the implementation of a strategic organizational committee for patient engagement in mental health
Source: PLoS One. 2018 Oct 24;13(10):e0205173. doi: 10.1371/journal.pone.0205173 (PMC6200221; doi:10.1371/journal.pone.0205173)
Supplement: S1 Appendix — (PDF) [file pone.0205173.s001.pdf]

S1 Appendix. The implementation process of the committee and influencing factors.

<table>
<tr><th></th><th>AGENDA-SETTING</th><th>MATCHING</th><th>REDEFINING/ RESTRUCTURING</th><th>CLARIFYING</th><th>ROUTINIZING</th></tr>
<tr><td>Content of implementation process</td><td>Elaboration of patient engagement within one department (policy, compensation, etc.)

Training for participation

Creation of self-help group

Introduction of citizen psychiatry (CP) and recovery (R) combination</td><td>Organization of civic forum

Field research visits to explore recovery and citizenship psychiatry (France inspiring)

Conceptualization of full citizenship vision

Mandate assigned for realization of patient engagement within the new vision</td><td>Establishment of committee for patient engagement

Researcher and member of initiating department as first members (chairs)

Inclusion of several departments, the user committee, two patient partners (one as coordinator)

Change of chairperson

Elaboration of mandate and role of committee

Budget from executive management</td><td>Elaboration of centralized system:
- Formal policy and procedures
- Request form
- Compensation form
- Tracking mechanism

Reflection on functioning participation

Removal of discriminating organizational structures

Communication and promotion</td><td>Ongoing application of procedures

Creation of evaluation mechanism

Increased participation (activities and patient partners)</td></tr>
<tr><td rowspan="8">Organizational and environmental factors (facilitating (+)/limiting (-))</td><td colspan="5">Executive management support (+)</td></tr>
<tr><td colspan="5">Collaboration with self-help group (+)</td></tr>
<tr><td colspan="5">Resistance towards participation (especially in the beginning) and stigma (-)</td></tr>
<tr><td rowspan="2">Mental health policy (MHAP 2005-2010) (+)</td><td colspan="4">Vision of full citizenship (+)</td></tr>
<tr><td>Obligation to evolve in research and innovative practices (+)</td><td colspan="3">Financial resources (+)</td></tr>
<tr><td rowspan="3">Management support in initiating department (+)</td><td>Leadership of key actors in executive management /research department (+)</td><td colspan="3">“Standard-bearers” of full citizenship vision (+)</td></tr>
<tr><td>Difficulty in sharing the vision (-)</td><td colspan="3">Supportive network (+)</td></tr>
<tr><td>International influence (+)</td><td>Shared leadership management/research (+)</td><td></td><td>Reform in healthcare system (-)</td></tr>
</table>
